# Predicting active site residue annotations in the Pfam database

**DOI:** 10.1186/1471-2105-8-298

**Published:** 2007-08-09

**Authors:** Jaina Mistry, Alex Bateman, Robert D Finn

**Affiliations:** 1 Wellcome Trust Sanger Institute, Wellcome Trust Genome Campus, Hinxton, Cambridge, CB10 1SA, UK

## Abstract

**Background:**

Approximately 5% of Pfam families are enzymatic, but only a small fraction of the sequences within these families (<0.5%) have had the residues responsible for catalysis determined. To increase the active site annotations in the Pfam database, we have developed a strict set of rules, chosen to reduce the rate of false positives, which enable the transfer of experimentally determined active site residue data to other sequences within the same Pfam family.

**Description:**

We have created a large database of predicted active site residues. On comparing our active site predictions to those found in UniProtKB, Catalytic Site Atlas, PROSITE and *MEROPS *we find that we make many novel predictions. On investigating the small subset of predictions made by these databases that are not predicted by us, we found these sequences did not meet our strict criteria for prediction. We assessed the sensitivity and specificity of our methodology and estimate that only 3% of our predicted sequences are false positives.

**Conclusion:**

We have predicted 606110 active site residues, of which 94% are not found in UniProtKB, and have increased the active site annotations in Pfam by more than 200 fold. Although implemented for Pfam, the tool we have developed for transferring the data can be applied to any alignment with associated experimental active site data and is available for download. Our active site predictions are re-calculated at each Pfam release to ensure they are comprehensive and up to date. They provide one of the largest available databases of active site annotation.

## Background

Enzymes play a considerable role in controlling the flow of metabolites within a cell; they catalyze virtually all of the reactions that make and modify the molecules required in biological pathways. Only a small number of residues within an enzyme are directly involved in catalysis and the structure and chemical properties of these residues (termed the active site) determine the chemistry of the enzyme. For this reason active site residues are highly conserved.

Pfam [1] is a database of 8296 protein families (as of Pfam release 20.0). Only ~0.4% of the sequences contained within the enzymatic Pfam families (i.e. those families that contain at least one characterized catalytic site) have the active site residues experimentally determined. There are families within Pfam which we know are catalytic, yet the residues that perform catalysis have not been characterized for any of the sequences within them, for example family YgbB (PF02542). Even where a structure is known, there are cases where the catalytic residues have not been identified (e.g. Swiss-Prot:P30085). Although the proportion of characterised catalytic residues known is low, many enzymatic sequences within a Pfam alignment are homologous to a protein whose catalytic residues have been characterised.

The fraction of characterized sequences continues to diminish as high throughput genome sequencing projects generate more and more data. To overcome the lack of experimental data we can use computational methods to predict functional residues on new protein sequences.

A range of approaches has been applied to the task of predicting active sites in protein sequences computationally. These can be split into two broad categories: those that transfer experimentally characterized active site data by similarity and those that predict active site residues *ab initio*.

The *ab initio *methods for catalytic site prediction exploit some of the known properties of active sites: active sites are usually found buried within a cleft of a protein, mutations in them can often increase the stability of an enzyme and they are highly conserved. This has led to the use of geometry data [2-5], stability profiles [4,6,7] and sequence conservation [8-11] in active site prediction. In addition, the different approaches can be used in combination. Evolutionary trace (ET) is one such method which first identifies the most highly conserved residues in related sequences, maps them onto the structure of the protein and then examines the structure for clusters of residues which could correspond to active sites or other functional sites [12]. ET has been applied in automated approaches that have been reported to predict active sites successfully for structures in 60–80% of test cases [13-15]. There has been some work on developing motif based methods to predict functional sites, however these have generally shown a high rate of false positives (FPs) [16-18]. Neural networks [19] and support vector machines [20,21] are other types of computational approaches which use structure and sequence information to predict active site residues. The different methods are hard to compare to each other in terms of accuracy since a range of tests have been used and in each case the tests are performed on a relatively small set of different enzymes (<200 structures in the case of the structural methods). However, it is clear that they all have a relatively high rate of FPs.

Similarity transfer based methods use tools such as BLAST searches, hidden Markov models (HMMs), pattern matching and structural templates to first identify sequences homologous to those with known active site residues, and then transfer active site residues from the characterized sequences to the uncharacterized sequences. The Catalytic Site Atlas (CSA) [22] is a database that collates active site residues from the literature for proteins with a known structure. It also provides active site residue predictions for proteins with a known structure which it infers on the basis of PSI-BLAST hits, and it is one of the largest resources for catalytic sites. Another database containing literature collated active site residues and predicted active site residues is UniProtKB [23], the central repository for protein sequences. UniProtKB is composed of two sections, the hand annotated 'UniProtKB/Swiss-Prot' section and the automatically generated 'UniProtKB/TrEMBL' section. UniProtKB however, currently only predicts active site residues by similarity for sequences in UniProtKB/Swiss-Prot, and not for the sequences in the automatically generated UniProtKB/TrEMBL entries which form ~94% of this database. Additionally, it can sometimes be difficult to trace the evidence for a particular active site prediction in UniProtKB. PROSITE [24] is a database that contains a collection of regular expressions (patterns) against which sequences can be searched. Each regular expression represents a conserved motif such as an active site region. Each PROSITE pattern is searched against UniProtKB/Swiss-Prot and the resulting matches are manually annotated by curators as true positives (TP), false positives (FP), false negatives (FN) or potential (P). PROSITE matches to UniProtKB/TrEMBL sequences are available via InterPro [25]. These matches are verified using a set of secondary patterns derived from the PROSITE pattern which are computed with the eMotif algorithm [26]. A stringent threshold of E = 10^-9 ^is used so that each eMotif pattern is expected to produce a random false positive hit in 1 in 10^9 ^matches. Based on the results of eMotif, UniProtKB/TrEMBL matches are annotated as 'true' or 'unknown'. Although not specifically designed for active site predictions, large scale PROSITE matches are available for UniProtKB sequences making them a useful resource for comparing our predicted data with.

Protein domain databases such as SMART [27] and *MEROPS *[28] also collate active site data from the literature and use sequence similarity based transfer to annotate active site residues onto the sequences in their protein families.

Pfam contains a large collection of protein alignments and is one of the leading protein domain databases in terms of sequence coverage; 74% of the sequences in UniProtKB have at least one match to a Pfam domain (statistics taken from Pfam 20.0). Pfam contains the experimental active site annotations present in UniProtKB. To enrich the sequence annotations in Pfam, we have taken known active site residues defined by UniProtKB that occur within a Pfam alignment and used them to predict active site residues on other sequences within the same alignment. Using this methodology we have created one of the largest databases of active site predictions. Here we outline our methodology for active site residue transfer and compare our prediction data to four other databases. We also estimate the specificity and sensitivity of our methodology.

## Construction and content

The manually curated thresholds for each Pfam family are chosen such that the family contains no known FPs, therefore all sequences within a family can be considered homologous [1]. The active site Pfam families can contain both active and inactive homologues. This gives us an initial starting point of an alignment of sequences that share a particular domain.

The Pfam flatfiles originally contained the active site residue annotations present in UniProtKB/Swiss-Prot. As authors of the Pfam database we noticed that within the catalytic Pfam families, very few sequences had active site residue annotations and within the large alignments, the known active site residues can easily be overlooked. Furthermore, if one looked at the known active site residues from UniProtKB/Swiss-Prot in a Pfam alignment, one could see that these residues are conserved in many of the sequences without active site annotation. The Pfam database is renowned for having no known false positives in its alignments, so, to keep in line with this philosophy, we have developed a set of rules that allows conservative transfer of active site annotation from one protein to another protein in the same Pfam alignment.

**Figure 1:**
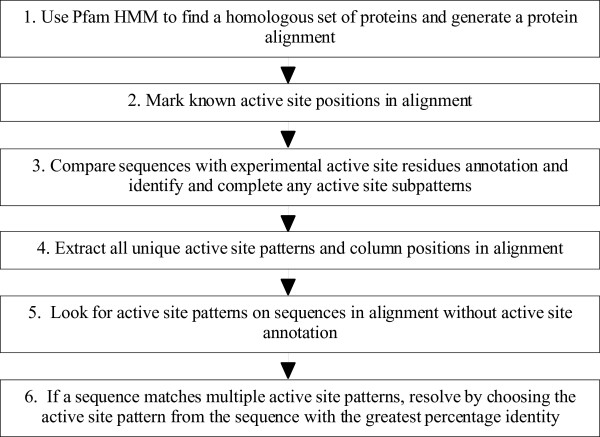
Flow diagram outlining the steps of the methodology we use to predict active site residues. Steps 1 and 2 are already present in Pfam and our methodology adds steps 3–6. See text for discussion.

To predict active site residues in a Pfam alignment we identify sequences with experimentally verified active site residues and use this information to predict active site residues in other members of that family. Our methodology is composed of a strict set of rules, which we have drawn up to prevent the transfer of active site annotation on enzymatically inactive homologues which may be present within Pfam families. Although we have applied it to Pfam alignments, the methodology works with any alignment and source of active site data. The logic of the rule based methodology is as follows and is outlined in Figure 1 (note that steps 1 and 2 are already present in Pfam).

**Figure 2:**
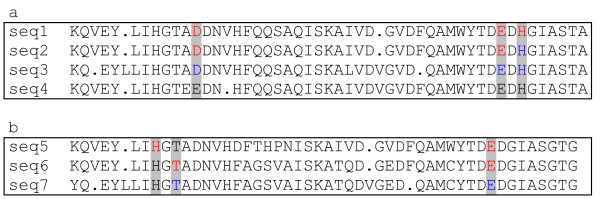
Two example alignments (a and b) showing scenarios in which residues are predicted to be active site residues using our rule based methodology. Residues in red denote an experimental active site, and residues in blue denote predicted active site residues. Grey columns indicated the presence of experimental active site data in the alignment, and these are the columns in the alignment which are methodology focuses on. See Construction and Content for details.

The first step is to use HMMs to find a homologous set of proteins and generate a protein alignment (Figure 1 step1). We identify the positions of all experimentally verified active sites in the alignment (Figure 1 step 2), and perform an all-against-all comparison of sequences within an alignment that contain experimentally determined active site residues (Figure 1 step 3). The comparison not only removes redundancy but also allows the identification of sequences for which only a subset of the active site residues have been experimentally determined. Figure 2 shows two alignments in which residues are predicted to be active site residues using our rule based methodology. In the first alignment (Figure 2a), sequence 1 contains three experimental active sites (D, E and H – the 'active site pattern') and sequence 2 contains two experimentally defined active site residues (D and E). Since sequence 2 has H aligned with the active site H of sequence 1 (and as both the D and E active site positions in sequences 1 and 2 align), the H in sequence 2 is predicted to be an active site residue in addition to the two experimentally defined residues. If similar patterns overlap but do not align then these are treated as separate distinct active site patterns.

Step 4 is to identify all the active site patterns and their column positions (Figure 1 step 4). For Figure 2a this is D in column 13, E in column 43 and H in column 45. Each unannotated sequence in the alignment is analyzed to see if it contains an exact match to the active site pattern (Figure 1 step 5). For example, sequence 3 contains the residues D, E and H in the active site residue columns (as found in sequence 1) and so these are predicted to be active site residues. Sequence 4 contains E and H in the active site residue columns, however it does not have residue D in column 13, so no active site residues are predicted. Although D to E in column 13 is a conservative substitution, the methodology currently does not make allowances for conservative residue substitutions at a particular position.

Finally (step 6) when there are two distinct experimentally determined active site patterns within a family, each unannotated sequence is compared as before. There are cases where an unannotated sequence matches more than one active site pattern. In such cases the active site residues from the sequence in the redundant set of active site patterns with the greatest percentage identity to the unannotated sequence are predicted. An example scenario of this is shown in Figure 2b. In this scenario sequence 5 contains two experimentally verified active site residues: H in column 9, and E in column 42. Sequence 6 contains two experimentally verified active site residues: T in column 11, and E in column 42. As before, a comparison of the experimentally verified active sites is performed. Note that although sequence 5 contains a T in column 11 and sequence 6 contains a H in column 9, these two patterns are not merged since neither is a subset of the other. Although one could argue that the true active site pattern for the family should be the union of the active sites of sequence 5 and sequence 6, we choose not to combine these patterns since the union of the two active site patterns has not been experimentally observed. Merging active site patterns would potentially increase our sensitivity but also result in an increase in FPs. Thus, in Figure 2b we have for predictive purposes two distinct, yet overlapping, active site residue patterns. Sequence 7 contains the active site patterns found in both sequence 5 and sequence 6. In this case sequence 7 has a higher percentage identity to sequence 6 than sequence 5, so only the T in column 11 and E in column 42 of sequence 7 are predicted to be active site residues, (the active site pattern from sequence 6).

In order to test the ability of these rules to predict active site residues, we used the 8296 alignments from Pfam 20.0 and the experimentally verified active site residues from two different databases, UniProtKB and CSA. Both resources provide predicted active site residues based on different methods. We compared the results of our predictions to each of the database predictions.

UniProtKB Knowledgebase (release 8.0) contains 2735 experimentally verified active site residues and a further 45698 predicted active site residues (these are annotated in UniProtKB as being 'by similarity', 'potential' and 'probable'). The underlying Pfam sequence database is based on UniProtKB, thus the active site residue positions from UniProtKB are easy to transfer onto the Pfam alignments of UniProtKB sequences.

The experimentally determined active site residues found in the CSA database are defined on proteins of known structure found in PDB. CSA also contains predictions for other sequences in PDB based on PSI-BLAST searches [22]. To allow the experimentally defined CSA dataset to be used with Pfam alignments, we converted the CSA (version 2.1.8) active site data from PDB residue positions to UniProtKB residue positions using the mapping provided by the MSD [29]. The resulting CSA dataset contains a total of 1495 literature active site residues and an additional 5517 predicted active sites residues. The experimental active site data was transferred to the alignments within Pfam using the methodology described above and our predictions were compared to those of CSA.

We also compared our active site data to that of the active site patterns found in PROSITE. We extracted the sequences matching the active site PROSITE patterns from InterPro 14.0 (based on PROSITE 19.30). InterPro annotates PROSITE matches in UniProtKB/TrEMBL as 'true' if a sequence match to a PROSITE pattern is confirmed by eMotif, and 'unknown' if it is not. For UniProtKB/Swiss-Prot sequences matches, PROSITE hits are available with manual annotation (TP, FP, FN or P). In our analysis we compared the number of sequences annotated as active site PROSITE hits (potential or TP/true) with the number of sequences in Pfam that had a Pfam active site residue. We also examined the sequences in UniProtKB/Swiss-Prot which matched an active site PROSITE pattern and compared the manual annotation of these sequences to our Pfam active site predictions.

To test the sensitivity and specificity of our method we compared our prediction data and that of PROSITE to the data in *MEROPS*. We defined a TP as a sequence on which there is a PROSITE hit (annotated as TP or true) or where Pfam predicted an active site residue in the peptidase region of a sequence defined by *MEROPS *as a peptidase, and defined a FP as a sequence that *MEROPS *defined as a non-peptidase homologue and where there was a PROSITE hit or Pfam predicted active site residue. We defined a TN as a sequence on which there were no PROSITE hits or Pfam active sites within the peptidase region of a sequence that *MEROPS *defined as a non-peptidase homologue, and a FN as a sequence on which there were no PROSITE hits or Pfam active sites but *MEROPS *defined the sequence as being a peptidase. The following equations were used to calculate specificity and sensitivity.

Sensitivity = TP/(TP+FN)

Specificity = TN/(TN+FP)

During our analyses we found that with using UniProtKB experimental active sites as our source of known active sites we obtained a low false positive rate. The UniProtKB experimental active sites are more comprehensive than the CSA in that they cover sequences with both known and unknown structure. For these reasons we have chosen UniProtKB as our preferred source of experimental active sites for Pfam (see Discussion for further details).

**Figure 3:**
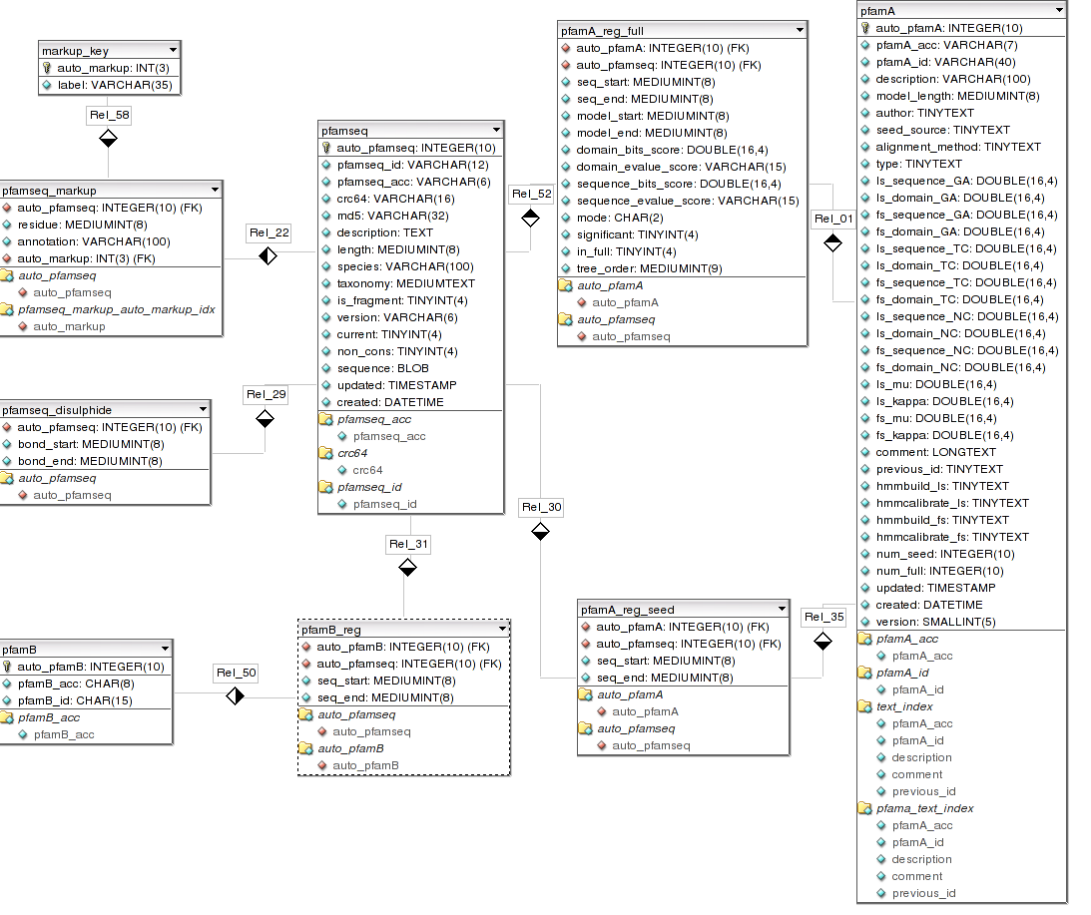
Database entities diagram showing the active site tables (pfamseq_markup and markup_key) in relation to other key tables in the Pfam database. The 'pfamseq' table is one of the central tables in Pfam and contains the sequence information. The Pfam-A alignments (both seed and full) and Pfam-B alignments that a particular active site residue belongs to can be accessed via the tables that link off pfamseq (pfamA_reg_full, pfamA_reg_seed, pfamA, pfamB_reg, pfamB). The red diamonds denote an indexed column and the key symbol denotes the primary key. The diamonds connecting the tables indicate many-to-one relationships with the dark filled end of the diamond indicating the many side of the relationship.

The active site data predicted with our methodology using the UniProtKB experimental data are re-calculated at each Pfam release. Each Pfam family has two associated alignments called the 'seed' alignment and the 'full' alignment (see [30] for further details). We run our methodology on the full alignments for each family, and use the resulting data to markup sequences in the seed alignment. To supplement our annotations, UniProtKB predicted active sites are stored/displayed if they are not predicted by our methodology. Our active site data is stored in a MySQL database and the schema is outlined in Figure 3.

## Utility

Our methodology predicts active site residues on sequences which are homologous to sequences with experimentally known active site residues. The data we have generated were originally intended to aid proteome annotation, but they will also be of interest to those working in fields such as comparative genomics, protein evolution and active site characterization.

We make our data available in a variety of different ways that should provide a suitable access point for a wide range of users. See Availability and requirements section for the address of the Pfam website and ftp site

**Figure 4:**
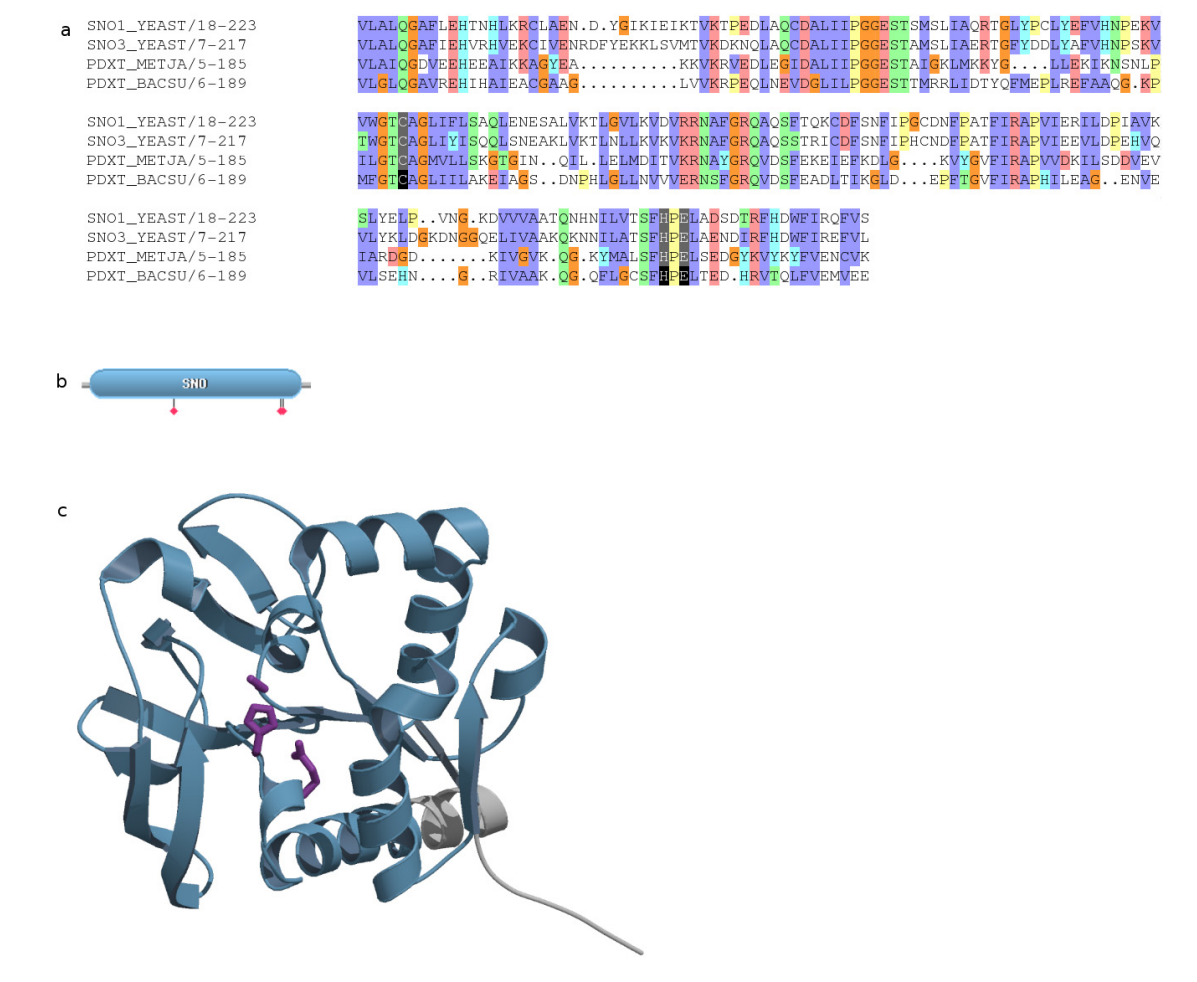
Sample multiple sequence alignment with active site markup in Stockholm format as used in the Pfam flatfiles. AS indicates an experimental active site, pAS indicates a Pfam predicted active site and sAS indicates a UniProtKB predicted active site.

1. ' Traditional' Flatfile – The Pfam flatfiles contain the multiple sequence alignments for each family with active site residues marked with '*' in Stockholm format. A sample alignment is shown in Figure 4. The flatfiles are also available for download via the ftp Pfam site.

**Figure 5:**
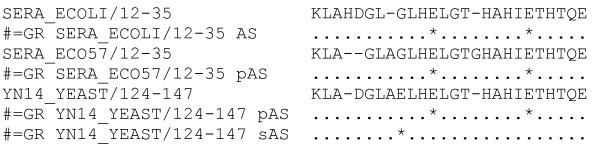
Sample multiple sequence alignment with active site markup in Stockholm format as used in the Pfam flatfiles. AS indicates an experimental active site, pAS indicates a Pfam predicted active site and sAS indicates a UniProtKB predicted active site.

2. Website – The Pfam alignments on the Pfam website highlights experimental active site residues with a black background. Our 'Pfam predicted' active site residues are shown on a dark grey background and UniProtKB predicted active site residues are marked with a light grey background (Figure 5a). The active site residues can also be viewed on the protein graphical domain view in which the experimentally verified active site residues are colored in red (Figure 5b), our Pfam predicted active site residues in purple, and UniProtKB predicted active site residues in pink. On the structural view of a protein we color the active site residues in a different color to the rest of the protein (Figure 5c).

3. DAS – Programmatic access to the data is available via a DAS features server that is written using ProServer [31] and provides the position and type of active site residues for the query sequence. See Availability and requirements section.

4. MySQL database – The Pfam MySQL database stores the location and type (experimental, predicted by Pfam or UniProtKB) of each active site residue, and is available for download from the Pfam ftp site.

The Perl code for implementing the rules detailed in the methodology is available and can be used with an alignment in either Stockholm or Selex format, and a file containing experimental active sites.

## Discussion

### Transfer of UniProtKB experimental data within Pfam alignments

**Figure 6:**
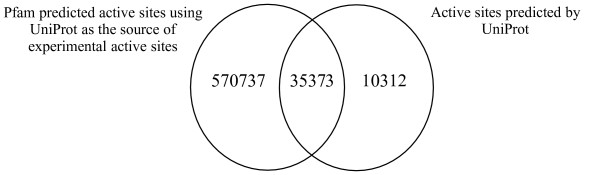
A Venn diagram comparing the active sites predicted by our methodology using the UniProtKB experimental active sites (Pfam predicted active sites) with the predicted active site residue annotation in UniProtKB.

Using the 2735 experimentally determined active site annotations in UniProtKB 8.0 and the alignments in Pfam 20.0, we have predicted 606110 active site residues. This compares to 45685 active site residues predicted by UniProtKB and increases the active site annotations in Pfam by more than 200 fold. Figure 6 shows the overlap of predicted active site residue annotation between our methodology, termed 'Pfam predicted', and UniProtKB. A significant proportion (35373 residues, 77%) of the active sites predicted by UniProtKB are also predicted by our methodology. To understand why we were unable to predict the remaining 23% (10312 residues), we investigated these sequences further.

**Figure 7:**
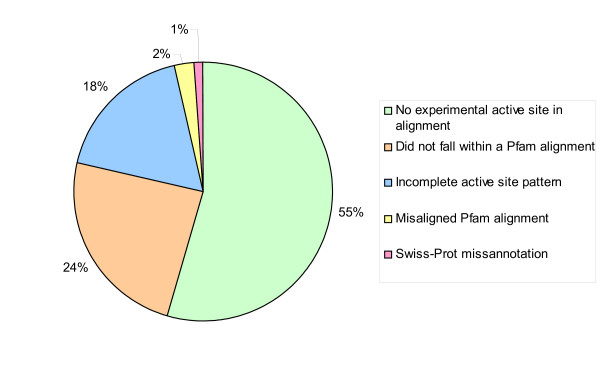
Breakdown of the UniProtKB predicted active sites that are not predicted using our methodology.

Of the 10312 UniProtKB predicted active sites that were not predicted by us, 55% (5601) were found in Pfam alignments that did not contain experimental UniProtKB active site residues at that position (Figure 7). The evidence that UniProtKB used to predict these residues is not transparent. We cannot predict these residues using our methodology because all of our predictions are based on transferring known experimental data within a Pfam alignment.

Another 24% (2501) of the UniProtKB predicted active sites that were not predicted by our methodology did not fall within a Pfam alignment. Again, these residues cannot be predicted since our methodology can only predict residues falling within the Pfam domain boundaries. The sequences which contain these active sites are cases in which the existing Pfam domain boundaries need extending, or are sources for potential new Pfam families. They have been flagged for the attention of the Pfam curators. 18% (1840) of the active site residues predicted by UniProtKB and not by Pfam were due to cases where the sequences in the family contained different residues at one or more positions, as compared to the active site residue pattern of the experimentally verified sequence(s). This is a drawback of our very strict rule based methodology since incomplete or conservative substitutions are not taken into account. A small proportion of cases were due to misalignments in the Pfam alignments (242 residues, 2%). Again these have been flagged for the attention of the Pfam curators. There were also some cases where UniProtKB predicted active sites were 'off by one' when compared to the experimental active sites and these appear to be cases where UniProtKB has mis-annotated a residue (128 residues, 1%).

A substantial proportion (96%, 570765 residues) of our active site predictions are not present in UniProtKB. This is due to the fact that unlike UniProtKB, which only makes predictions for sequences in UniProtKB/Swiss-Prot, we also make predictions for the automatically generated UniProtKB/TrEMBL entries. Comparing the active site residue prediction for UniProtKB/Swiss-Prot alone, our methodology predicts 48943 residues compared with the 45685 predicted by UniProtKB. Thus, we have 12570 additional active site predictions for the sequences in UniProtKB/Swiss-Prot. In the reverse comparison of UniProtKB against Pfam, UniProtKB only contains 6% of the active site information contained within Pfam.

### Transfer of CSA experimental data within Pfam alignments

**Figure 8:**
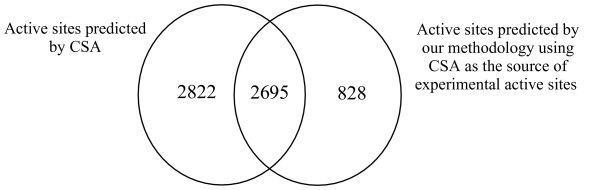
Venn diagram comparing the active sites predicted by our methodology using the CSA experimental active sites with the predicted active site residues annotations in CSA.

Next, we assessed our methodology by using the CSA experimental data. Figure 8 shows the comparison of the CSA PSI-BLAST predicted data and the data we generated (for sequences with a known structure) using the experimental CSA data (which they term 'literature'). There is some overlap (2695 residues) in our predictions, however the CSA predicts 5517 active site annotations compared with only 3523 predicted by our methodology. We analyzed the Pfam alignments that contain CSA predicted active site residues that are not predicted by our methodology, and found that in roughly half (1376 residues, 49%) of the cases there were no CSA experimental active sites within the Pfam alignments. These are cases where the experimental CSA active site sequence and the CSA predicted active site sequence are too divergent for both to belong to the same Pfam family.

To understand how our methodology compares to CSA when there is experimental data, we removed CSA predicted active sites where the predictions occurred in Pfam alignments that did not contain experimental active sites. After this filtering, there were still 1446 CSA predicted active sites that were not predicted by our methodology. These are cases where the criteria for predicting an active site are not met because one or more active site residues are different from the active site residues in the experimentally verified sequence. There are proportionally more of these cases here compared to when using the UniProtKB data due to the broader definition of an active site residue in CSA [22]. For example, in UniProtKB the sequence [Swiss-Prot:P77444] has residue 364 defined as an active site residue and residue 226 as a 'binding site' for pyridoxal phosphate, whereas CSA defines both residues 226 and 364 as active site residues. In addition, unlike our strict methodology, the CSA allows a one residue change per active site [22] when predicting active site residues.

We predicted 828 active site residues that CSA does not predict. These are cases where HMMER [32] (the package of programs used to construct the Pfam collection of HMMs) is able to identify homologous sequences that PSI-BLAST does not.

The narrower definition of experimental active site residues in UniProtKB works well with our conservative rule based methodology in that our prediction criteria are more often met than with the CSA experimental data. UniProtKB contains experimental active site annotations for sequences with known and unknown structure, and therefore has a greater number of active site sequences with associated experimental evidence than CSA. In addition to being able to predict a greater number of active site residues by using the UniProtKB data, it is easy to trace the source of our active site predictions since all the sequences in Pfam are present in UniProtKB. For these reasons we have chosen UniProtKB as our source of experimental active sites.

### Assessing sensitivity and specificity

In order to estimate the rate of false positives and to calculate the specificity and sensitivity (see equations 1 and 2 in Construction and Content) of our methodology we would ideally compare our data to an independent dataset. It is however difficult to find truly independent datasets since all of the resources utilise the active site annotation in UniProtKB.

We have chosen to compare our data to the active site data in PROSITE, because it is one of the most comprehensive, and to *MEROPS*, because it supplements its active site data with a thorough mining of literature data. In our calculations we assume that the predictions in these two databases are correct and compare our prediction data to theirs. We also compare the active site data of PROSITE to *MEROPS*.

#### Comparing Pfam to PROSITE

**Figure 9:**
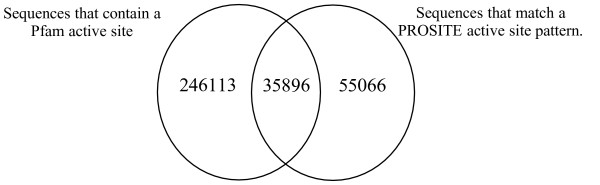
A Venn diagram comparing the number of UniProtKB sequences that contain a Pfam active site to the number of sequences that match an active site PROSITE pattern.

We compared the number of UniProtKB sequences that matched an active site PROSITE pattern to the number of sequences that contained a Pfam active site. Our method predicts approximately three times more active site sequences (282009) than PROSITE (90962), see Figure 9.

We examined the Pfam active site residues found on UniProtKB/Swiss-Prot sequences that had a match to an active site PROSITE pattern and compared them to the PROSITE manual annotation (TP, FP, FN, P) (see Table 1). We found that 29692 (99.5%) of our active site residues fell on a sequence region which matched an active site PROSITE pattern that was curated as a TP, 98 active sites (0.32%) were found on a PROSITE sequence region annotated as a FN, and only 44 active sites (0.15%) were present on a PROSITE sequence region annotated as a FP. We examined the 44 active sites that were FPs and found in each case there was good evidence (a strong alignment to a homologous sequence with experimental active site(s)) to annotate residues on these sequences as active site residues. Furthermore, in 5 of the 44 (11%) cases there was experimental evidence (in UniProtKB) to confirm that residues in these sequences were catalytic. This shows that the number of PROSITE matches manually annotated as FP are being overestimated.

**Table 1:** Comparison of sequences matching an active site PROSITE pattern with the manual annotation of these sequences by PROSITE

		Pfam active site predictions (%)	PROSITE active site predictions (%)
	TP	99.50	95.87
Manual annotation	FN	0.32	2.45
of PROSITE match	FP	0.15	1.36
	Potential	0.00	0.32

Of the PROSITE active site sequences in UniProtKB/Swiss-Prot, 136000 (95.87%) had been manually annotated as TPs, 3477 (2.45%) annotated as FNs, 1933 (1.36%) annotated as FPs and 450 (0.32%) were annotated as potential. This means that for the subset of UniProtKB/Swiss-Prot sequences for which we have manual annotation, our method has a higher sensitivity (99.18%) than PROSITE patterns (98.12%). (For sensitivity see equation 1 in Construction and Content).

We compared then compared *MEROPS *[28] active site data to both our data and PROSITE data. *MEROPS *is the definitive peptidase resource and uses literature data on active site residues and metal binding residues, sequence similarity, and manual analysis of alignments to classify peptidases and non-peptidase homologues into families. A sequence is considered to be a peptidase if it contains all the active site and (where appropriate) metal binding residues known for the family to which it belongs and a non-peptidase homologue if it lacks any one of these residues. We took the set of 22158 sequences that were common to both Pfam and *MEROPS*, and the 25903 sequences common to *MEROPS *and the PROSITE matches, and analyzed them to see if the two methods predicted active site residues/patterns on the peptidases but not on the non-peptidase homologue sequences.

#### Comparing Pfam with *MEROPS*

13779 (51%) of the sequences common to Pfam and *MEROPS *had Pfam active site predictions and were classified by *MEROPS *as being peptidases (TP). Only 900 (3%) of the sequences containing active site predictions as determined by our methodology were classified as non-peptidase homologues by *MEROPS *(FPs). This proportion is low compared to the documented FP rates outlined in the Background section. The set of FPs is comprised largely of sequences for which *MEROPS *identified a greater number of functional residues than UniProtKB. This means that if a sequence in a Pfam alignment contains an active site residue pattern as defined by UniProtKB, but is missing any additional sites that are classified as experimental active site residues or metal binding residues in *MEROPS*, it will be predicted to have active site residues using our methodology but *MEROPS *will classify it as a non-peptidase homologue. For example, sequence [Swiss-Prot:Q9YVR4] is predicted to have an active site residue (E376) using our methodology. *MEROPS *has identified this sequence as a non-peptidase homologue because, even though it contains the active site residue, it is missing one of the metal binding residues (H385). The lack of comprehensive data available for metal binding and/or substrate binding sites prevents us from including this data as a discriminator in our methodology. Despite this, our methodology still shows high specificity as demonstrated by the low proportion of FPs.

3860 (14%) of the sequences common to Pfam and *MEROPS *were classified in *MEROPS *as non-peptidase homologues and had no active sites predicted using our methodology (TNs). The remaining 8379 (31%) sequences were those where our methodology did not predict an active site but *MEROPS *classified them as being true peptidases (FNs). In these cases UniProtKB did not define any experimental active sites in the corresponding Pfam alignments and hence there is no information from which our methodology can infer active site information. This gives our data a specificity of 82%, and a sensitivity of 62%.

**Figure 10:**
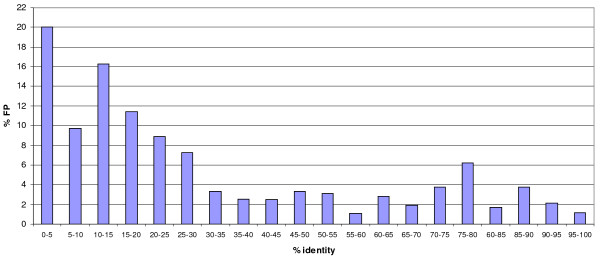
Fraction of Pfam predicted active site sequences that are false positives plotted against percentage identity to the nearest homologue with active site data within a Pfam alignment.

We took the set of Pfam predicted active site sequences and for each sequence, we calculated the percentage identity to the nearest homologue with experimental active site data in the corresponding Pfam alignment. We found there were a few cases where a FP sequence can have a sequence identity of >90% to a catalytically active sequence, yet because one or more functional residues are missing, it is catalytically inactive. Figure 10 shows the fraction of Pfam sequences that are FP (as identified from the *MEROPS/*Pfam comparison) against percentage identity. The FP set of sequences has a mean sequence identity of 23.6% while the TP distribution is shifted slightly to the right and has a mean of 32.3% (data not shown). Figure 10 shows that as percentage identity decreases, the chance that we have incorrectly predicted active sites on a sequence increases. Sequences with >30% identity have a lower likelihood of being a false positive. This means the rate of false positive predictions could be decreased by restricting transfer of active site annotation to sequences that have a percentage identity >30%.

#### Comparing PROSITE with *MEROPS*

To complete the comparison we compared the *MEROPS *data to that of active site PROSITE patterns. Only 1212 (5%) of the sequences common to *MEROPS *and PROSITE matched a PROSITE active site pattern and were defined in *MEROPS *as a peptidase (TPs). A large proportion of sequences (19979, 77%) were defined in *MEROPS *as a peptidase, but did not match an active site PROSITE pattern (FNs), which shows PROSITE patterns have a poor coverage of peptidase enzymes.

4532 (17%) of the sequences did not match a PROSITE pattern and were defined as a non-peptidase homologue in *MEROPS *(TNs). 180 (0.7%) sequences matched a PROSITE active site pattern but were defined as a non-peptidase homologue in *MEROPS *(FPs) which shows that of the active site sequences PROSITE does predict, it does so accurately. This gives the PROSITE peptidase active site patterns a high specificity of 96%, but a poor sensitivity of 6%.

## Conclusion

Our automated rule based methodology allows us to accurately transfer active site annotation between sequences within a Pfam alignment and other members within the same Pfam family. Using this methodology we have substantially increased the number of active site annotations in Pfam. Our active site data are available through a variety of methods (see Utility for further details on how to access our prediction data). The tool we have   developed for predicting active site residues is also available for   download (see Availability and requirements).

On comparing our predicted active site data to the UniProtKB predicted data, we found we are predicting significantly more active sites. When we compare both CSA predictions and UniProtKB/Swiss-Prot predictions to our active site predictions, we find that the sets overlap, but each contains additional novel predictions. Ultimately, the source of experimental data (which is different for UniProtKB and CSA) determines the success and coverage of any method that uses similarity for transferring active site information. We have chosen UniProtKB over CSA as our source of experimental active sites as it includes both sequences with and without known structures. Since Pfam alignments are built from UniProtKB sequences, each of our active site predictions can be easily traced back to the experimental reference sequence.

On comparing our data to PROSITE patterns we find our methodology detects three times more active site sequences. Within the UniProtKB/Swiss-Prot portion of UniProtKB (for which there is manual annotation for all of the PROSITE matches), we find our predictions have a lower FP rate (0.15%) than PROSITE patterns (1.36%).

The comparison with the *MEROPS *data showed our methodology to have a low FP rate (3%), a good specificity (82%), and a reasonable sensitivity (62%). This suggests our conservative, automated methodology allows us to confidently predict a substantial number of active site residues at the expense of losing some sensitivity. Investigating the percentage identity showed that below 30%, there is an increased chance of mis-predicting an inactive sequence as being catalytically active. This means we could further increase the specificity of our method by only transferring annotation to sequences that are >30% identical at a cost to sensitivity.

Our methodology fails for sequences which contain the catalytic residues but are missing any other residues that are essential for catalysis. Incorporating additional data such as the presence of metal binding and substrate binding residues into our methodology could improve our specificity, however this data is not consistently available for all sequences. The necessity for metal or substrate binding residues can also be particular to each mechanism of catalysis and hence is difficult to encapsulate in a simple set of rules.

In addition to finding new active site residues, the methodology draws attention to sequences that are members of an enzymatic family but which do not contain the active site residue patterns shared by enzymatic members of the family. This subset includes sequences which have a similar but non-identical amino acid at an active site position. In these cases the user is able to inspect the alignment along with other evidence in order to make a more informed judgement on its activity. The subset also includes non-enzymatic homologous sequences and novel enzymatic subfamilies that should be prioritized for biochemical characterization.

The catalogue of Pfam entries has a relatively high coverage of UniProtKB sequences which allows our active site prediction data to be comprehensive. New sequences that are added to UniProtKB are incorporated into Pfam at each release, and, by re-calculating our active site predictions, we ensure our data are regularly updated. As Pfam continues to grow through the addition of new families and the expansion of existing families, we expect the number of active site predictions to increase. The forthcoming release of Pfam 22.0 contains 100,000 more Pfam active sites than Pfam 20.0. Our active site dataset is the largest single resource of active site annotation currently available.

## Availability and requirements

The Pfam website from is accessible from .

The Pfam flatfiles, MySQL database and the Perl script that implements the rules of the methodology are available from the Pfam ftp site (see ,  and  respectively). Note that the Perl script requires BioPerl 1.4 to be installed. The Perl script is freely available and can be redistributed or modified under the terms of the GNU General Public License.

Programmatic access is available via the DAS features server . To access the data, use the UniProtKB accession in the form , where P00784 is the UniProtKB accession.

## Abbreviations

Catalytic Site Atlas (CSA), Evolutionary Trace (ET), Distributed Annotation System (DAS), hidden Markov model (HMM), true positive (TP), false positive (FP), true negative (TN), false negative (FN), potential (P)

## Authors' contributions

JM did the experimental work. JM and RDF devised the methodology and wrote the manuscript. AB provided guidance and critically reviewed the manuscript.
